# Does agricultural ecology cause environmental degradation? Empirical evidence from Bangladesh

**DOI:** 10.1016/j.heliyon.2022.e09750

**Published:** 2022-06-18

**Authors:** Shanjida Chowdhury, Sunjida Khan, Md Fouad Hossain Sarker, Md Kabirul Islam, Maruf Ahmed Tamal, Niaz Ahmed Khan

**Affiliations:** a Department of General Educational Development, Daffodil International University, Dhaka, Bangladesh; b Department of Business Administration, Daffodil International University, Dhaka, Bangladesh; c Department of Development Studies, Daffodil International University, Dhaka, Bangladesh; d Department of Multimedia and Creative Technology, Daffodil International University Dhaka, Dhaka, Bangladesh; e Division of Research, Daffodil International University, Dhaka, Bangladesh; f Department of Development Studies, University of Dhaka, Dhaka, Bangladesh

**Keywords:** Environmental degradation, Agricultural ecology, CO_2_ emission, Bangladesh

## Abstract

Agricultural sector accelerates a nation’s economic growth towards sustainable development. There exists a significant relationship between agriculture and the environment. Sustainable agricultural development ensures food quality and in tandem prevents natural calamities like drought. However, in order to fulfill the food demand of a growing population, poor law quality and untenable agriculture practices arise, which in turn lead to environmental degradation. The current study explores the relationship between the agro-economic atmosphere and CO_2_ emissions as a measure of environmental degradation in Bangladesh between the years of 1985 and 2017. To exhibit the long-run relationship of agricultural ecology and carbon dioxide emissions, three cointegrated equations- Fully-modified ordinary least square (FMOLS), Dynamic ordinary least square (DOLS), and Canonical cointegrated regression (CCR) were assessed. For cointegration, Bayer-Hanck cointegration was implied. In long-run estimates, it was found that livestock, rice area harvested, cereal production, and other crop production impeded environmental dilapidation. The Granger Causality Test enabled unidirectional causality towards burned biomass (crop residues), the agricultural economy, and carbon emissions. Therefore, this dimension’s causality concluded that carbon dioxide emissions were caused by cereal production, other agricultural production, and agricultural land production. The overall findings of this study could potentially assist the Government of Bangladesh and the necessary authorities for implementing synchronized policies to help reduce environmental pollution and set an example for other developing nations like Bangladesh.

## Highlights

• The relationship between agro-economic climate and CO_2_ emissions in Bangladesh from 1985 to 2017 is analysed
• The results exhibit that production of cereals, other agricultural products, and land production, increased CO_2_ emissions.
• Unplanned, static crop intensity and a lack of modern farming infrastructure degrade the environment.
• The findings may help Bangladesh achieve UN-SDG targets 7, 9, 12, and 13.

## 1 Introduction

Environmental degradation arises mainly due to the continuous emission of greenhouse gases from anthropogenic activities [1]. The Rio (1992) Earth Summit inferred that no credible alternative exists apart from emphasizing social, economic, and environmental dimensions to save the earth, and these three factors were closely interdependent with each other [2]. In order to meet a burgeoning population, modern technologies manipulate the ecological atmosphere, thereby leading to environmental degradation. Advanced technologies such as biomass have a negative impact on the environment [3]. Additionally, using modern technologies leads to consequences such as deforestation, land degradation, soil erosion, water pollution, air pollution, etc. which in turn affect farming and farm produce [4]. Since population is one of the most important determinants of demand, a continuous increase in the world population increases the worldwide demand for food to meet basic human needs [5].

**Figure 1:**
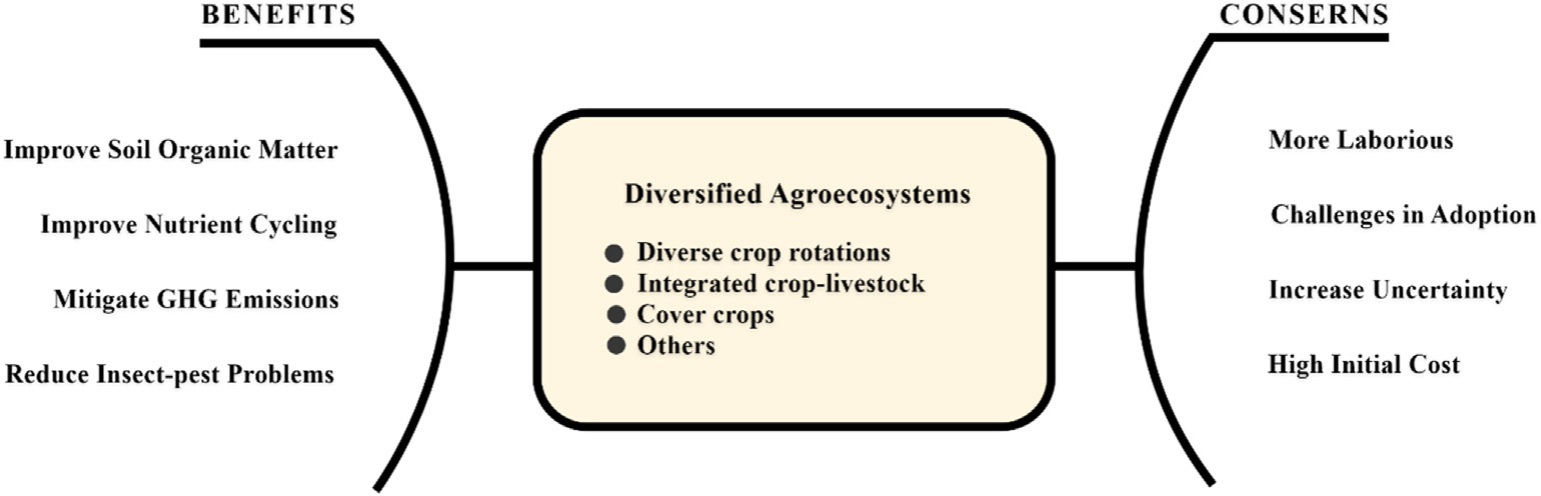
Benefits and concerns of diversified agroecosystems [*Source*: FAO AQUASTAT (2009)].

Agriculture is a leading form of global land management and the agricultural ecosystem covers nearly 40% of the land surface on earth [6]. Agricultural ecosystems are significantly limited to rural areas, with almost 50% of the world’s population living in these rural areas. Bangladesh is no different with approximately 70% of the population living in villages and depend on agriculture for their livelihood [7]. In many developing countries, agriculture is considered as the primary driver of economic growth, with almost 75% of agricultural value addition being produced in these nations [8]. Agro-ecosystems are the natural processes or ecosystems on agricultural land which have been altered to sustain the production of agricultural products such as food, harvestable goods, and fiber [9]. It is designed by humans and is used for enhancing agricultural production. Rapid food and fiber production are also considered as the causal factor for environmental degradation [10] (see Figure 1).

To ensure environmental quality for survival, it has been emphasized to maintain the same through sustainable and appropriate use of available resources. Land, water, mining, soil, forests, etc. are considered some common natural resources on which all agricultural activities rely on. Bangladesh has a rapidly expanding population and must produce agricultural products quickly and import from other countries to meet their needs [11]. Technology, as well as the judicious utilization of chemicals, biomass, and other materials would aid in the development of speedy results. Agricultural operations such as land reclamation, irrigation, crop breeding, and deforestation among other things end up polluting the environment [12]. Rapid farming activities may cause soil degradation, soil erosion, air pollution, deforestation, desertification, and many other negative effects, which in turn would affect farming and farm produce [12]. Increased pesticide usage harms farmworkers via extended exposure times and contaminate ground and surface water sources, including harming downstream users and inland fisheries [13]. According to a recent World Bank report, Bangladesh is one of the most densely populated countries in the world [14]. This has causes a significant rise in the demand for diverse agricultural products which has resulted in continuous greenhouse gas (GHG) emissions. According to a report by Climate Watch [15], GHG emissions in Bangladesh increased significantly and reached a historic high of 220.75MT CO_2_e in 2018. The increase in emissions was mainly driven by agriculture (88.53MT) followed by Energy (85.84MT), Land-Use Change and Forestry (21.78MT), Waste (20.64MT) and Industrial Processes (3.97MT). Though meeting the requirements of a rising population is vital, a healthy environment is also a significant requirement for human survival [16]. Based on the review of past research, it was concluded that more insight into the relationship between agricultural ecology and environmental degradation in Bangladesh was needed. Numerous studies have been conducted in Bangladesh focusing on environmental degradation, but the relationship between environmental degradation and agricultural ecosystems has received little to no attention.

Based on the aforementioned points, the major objective of this study was to investigate the nexus between Bangladesh’s agro-economic environment and CO_2_ emissions as a measure of environmental degradation from 1985 to 2017.

## 2 Literature review

Climate change is the most pressing issue facing the entire globe today. Anthropogenic greenhouse gas emissions contribute to global warming, consequently causing long-term impact on weather patterns and ozone layer depletion. Agriculture services such as land erosion control, irrigation water management, insect infestation control, boosting nutrient cycling, pollination, and others are utilized to increase harvestable product yield. However, these processes interfere with the natural cycle and are therefore considered detrimental due to causing water loss and soil quality reduction amongst other harmful consequences [17].

Agriculture operations have a net negative impact on overall environmental quality [18]. The waste produced during these activities pollute the soil, water, and environment. A past study discovered a positive association between agricultural productivity and ecological pressure, i.e. as the product grows, so does the demand on the environment. Following the Green Revolution, synthetic fertilizers and pesticides have accounted for a substantial percentage of agriculture dependency [19]. Rapid food production with synthetic fertilizers is preferred, not only because of the importance of agriculture but also to alleviate food safety concerns. Consequently, it has caused hazards for human health and the surrounding survival environment. Another past study [20] identified a U-shaped relationship between carbon dioxide emission (CO_2_) and agricultural development. The analysis was based on the agriculture sector in Ghana and recommended some policy suggestions emphasizing large-scale adoption of environment-friendly techniques for reduction of emissions and concurrent agricultural development. It has also been noted [21] that wetland rice production acted as an important contributor in reducing greenhouse gas (GHG) emissions. The concerned study indicated that non-puddling rice production with increased residue retention reduced more GHG emissions in comparison to non-puddling rice production with decreased residue retention. Therefore, high residue retention contributed to increased emissions removal. Another study revealed a positive relationship between carbon dioxide emissions (CO_2_) and agricultural development, leading to policy recommendations that focused on large-scale adoption and ecologically acceptable approaches for decreasing emissions and advancing agricultural development [20]. Past studies have shown that puddling with lower residue retention during rice production reduces GHG emissions more when compared to non-puddling methods [21]. Consequently, it can be stated that high residue retention has a greater influence on decreasing emissions.

Rahman [22] performed a study to establish an environmentally beneficial technique known as ‘Environment Smart Agriculture’ (ESA). The study used an indicator to exhibit that using chemicals had a positive impact on agricultural produce’s long-term viability and also stated that there had to be an improvement in the farmers' understanding of the use of chemicals in agriculture. Another study [23] devised an indicator for determining the level of environmental degradation. The results of the study were indicative that the production of HYV rice caused a 27–69% increase in the theoretical maximum amount of environmental harm, alluding to substantial environmental deterioration and thereby raising worries among policymakers. According to a study by Faroque [24], the indiscriminate usage of chemicals in agricultural production caused environmental deterioration, which in turn posed a threat to agricultural viability. The government has been attempting to promote on-farm resources and encourage alternative usage of external inputs and pesticides. The study stressed the importance of reducing chemical use in agriculture.

Higher economic growth is dependent on the extensive use of resources to sustain demand, and this can create serious environmental pollution. Thus, it can be stated that there is a trade-off relationship between environmental pollution and economic growth. Under-developed and developing nations mostly depend on agriculture for boosting their economy. As and when their agricultural productivity increases, more resources tend to be used, thereby leading to environmental degradation [25]. It has been exhibited that the consumption of natural resources via mining, agriculture, and deforestation has the potential to degrade environmental quality by increasing emissions [26]. The ambition to expand the agricultural frontier in Livelihood Adaptation to Climate Change (LACCs) where agricultural activities are dominating leads to damaged vegetation, desertification, and deforestation, exposing the land to deterioration [27].

Nathaniel and Adeleye [28] showed that the UN-SGDs (SDGs-9) emphasized the need for reducing carbon emissions and preventing environmental damage. Carbon emissions significantly contribute to environmental degradation, thereby posing a challenge to establishing and maintaining a sustainable ecosystem. The authors also studied a variety of factors that influenced CO_2_ emissions and their potentially harmful consequences on the ecosystem. Usman et al. [29] discovered a substantial link between agricultural growth, economic growth, and the use of non-renewable energy, which caused environmental degradation. The study also found a link between carbon emissions and economic growth, which was a roadblock to meeting sustainable development targets.

According to Hafeez et al. [30], agriculture was responsible for 21% of global CO_2_ emissions, and concluded that agriculture and energy consumption were driving environmental degradation. They also emphasized energy efficiency and use of eco-friendly technology. Olanipekun [31], stated that growing population and poverty had a significant impact on environmental degradation in emerging countries. As the population grows, so does the demand for food, necessitating massive food production in a short period of time. Consequently, agricultural practices relying on rapid output may be unsustainable and negatively impact the environment due to CO_2_ emissions.

The natural climate system’s balance is usually stabilized by the local ecosystem, including carbon, nitrogen, and water cycles. Greenhouse gases have been shown to be the long-term sources of climate change [32]. Global warming results from increased greenhouse gas emissions and it has the potential to alter the environment over time [33]. CO_2_, F, CH_4_, and N_2_O are the main greenhouse gases found in anthropogenic emissions and among them, CO_2_ contributes to nearly 76%, which causes global warming [34]. Environmental degradation has been of concern worldwide over the previous decades [35], whereas and the agricultural sector accounts for 10–15% of global emissions [36]. Even though developing countries heavily rely on agriculture, rising CO_2_ emission levels have been shown to reduce land productivity and inversely affect agricultural output [37].

CO_2_ emissions have adversely changed the climate, thereby disrupting the agro-ecological balance and income distribution, which creates insecurity in food consumption [38]. Agricultural greenhouse gases are mainly created from the cultivation of rice, crop residue burning, and livestock. Large volumes of GHGs are emitted during agricultural production mainly due to overexploitation of agricultural resources such as land, usage of chemical fertilizer and pesticides, ultimately resulting in climate change. Additionally, studies have also noted that rising food production to feed the world’s growing population has resulted in inefficient farming methods that inevitably degrade the natural environment [39]. Agriculture has also been shown to be significantly impacted by environmental challenges such as climate change, global warming, agricultural land degradation, water and air pollution, and biodiversity loss. Thus, future expansion of food output must be balanced against the growing scarcity of natural resources [36]. Studies have also shown that CO_2_ emissions from agricultural sources account for approximately 21% of total anthropogenic GHG emissions [38]. As a result of changing agro-ecological conditions, environmental degradation ultimately reduces agricultural output, leading to unequal income distribution which in turn leads to food insecurity [38].

Sustainable Development Goals (SDGs) formulated by the United Nations is a global policy action for all nations to implement in order to promote prosperity while simultaneously protecting the environment. Accordingly, ensuring a poverty-free world by the year 2030 forms the first goal of the SDGs [36]. The SDGs aim to increase agriculture productivity and ensure stable food security for all people by promoting sustainable consumption, production, and distribution approaches. Consequently, agriculture forms a vital issue for improving the quality of life and achieving the SDGs [40]. It is considered to be possible to reduce the inverse effect of agriculture on the environment by adopting strong and appropriate policies and technological transmission [41, 42].

According to Roberts [43], human communities have created a threat and negatively changed the global ecosystem via increased CO_2_ emissions. The increase in the population and animals of developing nations increases the production of food and livestock and thereby leads to increased emissions [44]. To meet the global population’s food demand, the agriculture sector has been under enormous strain and has produced emissions [45]. Sustainable agriculture helps increase food production while concurrently reducing the use of pesticides. Agriculture production has also been observed to accumulate carbon at times, thus polluting the environment [46]. It has been shown that there is a significant relationship between economic growth and environmental pollution. On the other hand, pollution reduction and environmental balance are not prioritized enough in developing and underdeveloped nations [47, 48].

### 2.1 Agriculture and environment in Bangladesh context

Bangladesh is primarily an agricultural country and the agricultural industry plays a vital role in driving the economic growth of the country. Agriculture employs over half of the Bangladeshi population, with more than 70% of the country’s land dedicated to agricultural production [49]. According to the report [50], agriculture's contribution to the GDP of Bangladesh grew to 11540.50 million BDT in 2021 from 11242.30 million BDT in 2020. However, the agricultural sectors (e.g., rice cultivation, enteric fermentation, synthetic fertilizers, livestock manure etc.) contributed to about 28% of total GHG emissions in 2018 [51], thereby posing a huge environmental concern. Similar findings were obtained in an earlier study [52], where the authors found a significant relationship between agricultural development and CO_2_ emissions. The research also showed that population, agriculture energy intensity and the economic activities were the factors most responsible for the increase in CO_2_ emissions which negatively impacted agriculture as well as the environment. In another study [53], the environmental consequences of Green Revolution technology diffusion in Bangladesh was explored. The study used 21 villages in three agro-ecological zones in Bangladesh as the sample area and factors like loss of soil fertility, trends in fertilizer and pesticide productivity at the national level, as well as farmers' awareness etc. were selected and examined as evidences. The results of the study indicated that Bangladesh had lost soil fertility in 11 out of its 30 agro-ecological zones to the tune of 10–70% between 1968 and 1998 due to intensive crop cultivation practices. A later study [54] revealed a casual nexus between agricultural output and CO_2_ emission levels wherein empirical evidences revealed that the agricultural output of Bangladesh was not a Granger causal for CO_2_ emissions, but the country’s CO_2_ emissions were a Granger causal for its agricultural output. Similarly, in another study [55], the authors identified that enormous population, economic factors, and human activities of low-income level nations like Bangladesh generated increased levels of CO_2_ emissions. Currently, Bangladesh contributes to around 0.14% of the world's CO_2_ emissions.

Using the cointegration technique, this study proposes to investigate the causal relationship between agricultural ecosystems and environmental degradation. To the best of the authors’ knowledge, none of the past Bangladesh centric studies have examined the causal relationship between agricultural ecosystems and environmental contamination. This study envisages to be a major step forward in environmental and agricultural research.

## 3 Materials and methods

This study investigated the linear relationship between environmental degradation and agricultural ecology in the period of 1985–2017 in Bangladesh by using annual datasets. Data, except for Bangladesh’s carbon dioxide emissions, was collected by the FAO [56]. Due to the lack of availability of most recent data, this study was limited to data available until 2017. Table 1 highlights the description alongwith a variable name. Various studies have shown that biomass-burned crop residues [57], agriculture value-added [58], CO_2_ equivalent to N_2_O emissions from synthetic fertilizers [59], livestock stock [60], agriculture machinery [60], cereal production [60], all have a significant impact on greenhouse gas emissions. Based on these past studies, all of these variables were used as explanatory variables in this study.

**Table 1:** Definition of variables under econometric analysis.

Variable	Description
CO_2_	Log of carbon dioxide emissions (Kt)
BM	Log of biomass-burned crop residues (dry matter; tons)
AG	Log of agriculture value added (% of GDP)
SF	Log of emissions of CO_2_ equivalent of N2O from synthetic fertilizers (Gg)
LS	Log of stock of livestock (number of head)
MC	Log of agricultural machinery (number of tractors)
RI	Log of rice area paddy harvested (Ha)
CP	Log of cereal production (tons)
OT	Log of other crop productions (tons)

The Intergovernmental Panel on Climate Change (IPCC) adopted the Tier 1 approach for measuring CO_2_ emissions due to climate change. All member countries estimate their CO_2_ emissions under the guidance of this strategy [61]. The current study calculates this in kilotons (Kt) and as a dependent variable, and the remaining variables treated as independent variables. The value added to agriculture was calculated in terms of its percentage share of the GDP. Crop residues, cereal production, and other crop production were all taken into account and their units are represented in tons. Synthetic fertilizer emissions were quantified as gigagrams of CO_2_ equivalent (N_2_O). In the case of livestock capacity, the head number was counted for this study. Machinery and tractors used for agricultural purposes were calculated in raw numbers. Since rice is the core factor of agricultural production in Bangladesh, paddy harvested per hectare was considered for this study's purposes. In order to avoid multicollinearity, all variable data was converted to natural logarithmic form and the following regression was provided to exhibit the relationship between carbon emission and the explanatory variables.

$$CO_{2}=f\left(BM,\;AG,\;SF,\;LS,\;MC,\;RI,\;CP,\;OT\right)$$

For exhibiting this linear combination, the equation was converted into the log-linear model. The final regression model for employing multiple cointegrated relationships is represented as follows:

$$ln{\left(CO_{2}\right)}_{t}=\beta_{0}+\beta_{1}lnBM_{t}+\beta_{2}lnAG_{t}+\beta_{3}lnSF_{t}+\beta_{4}lnLS_{t}+\beta_{5}lnMC_{t}+\beta_{6}lnRI_{t}+\beta_{7}lnCP_{t}+\beta_{8}lnOT_{t}+\varepsilon_{t}$$

In this final equation, ${\text{β}}_{0}$ is the constant, ${\text{β}}_{1}$ to ${\text{β}}_{8}$ are the parameter coefficients of respected variables and ${\text{ε}}_{\text{t ​}}$ is the stochastic error term. To anticipate the direction or effect of variables on carbon emission, it is dependent on the sign of $\beta_{i}$’s. for more granularity, biomass residues accelerate environmental degradation if $\beta_{1}$ >0 and vice versa if < 0. The agricultural output would increase if $\beta_{2}$ >0 through the positive impact of carbon emission. Similarly, synthetic fertilizer and the amount of livestock would increase if $\beta_{3}$ and $\beta_{4}$ are positive. For exhibiting the proxy of modern technology on agricultural production, this study takes into account agricultural machinery as an explanatory variable. If the impact of the same is positive, then $\beta_{5}$ > 0, otherwise it decreases while being negatively impacted. To check the robustness of agricultural output, three more variables on crops-related production were employed for this study viz. production of principal food rice, cereal or crop and miscellaneous crops production. The linkage between environmental disaster and these three variables would be positive if $\beta_{6,\;}\beta_{7}$, and $\beta_{8}$ are individually positive.

We used FMOLS, DOLS, and CCR estimators to demonstrate long-term behavior. These three estimators are emphasized for the following reasons. Firstly, though the OLS estimator is super-consistent [62, 63], the estimated t-statistic becomes approximately normal at normal level or stationary. Moreover, the bias convergence turns low in finite samples. Secondly, the t-statistic becomes insignificant in the case of omitted dynamics captured by residuals and it suffers from serial correlation and heteroscedasticity. Thirdly, FMOLS and DOLS have specific criteria. FMOLS works on the I(1) series [64], rectifies endogeneity and serial correlation effects [65], while DOLS is employed to estimate long-run equilibrium which is corrected for potential simultaneity bias among regressors [65]. Finally, DOLS is more useful to Johansen’s cointegration, as compared to FMOLS, or CCR. Being a robust but single equation method, misspecification error does not arise [62]. Moreover, DOLS has no demanding restriction on the order of variables, given that regressors should be integrated in order (1). DOLS regresses one of the I(1) variables on other I(1) variables, I(0) variables, and lags and leads to the first differences of the I(1) variables.

Unit root tests are considered important for performing time series analysis when arranging variables in a stationary order. This study employed a version of the unit root test known as Augmented-Dickey Fuller test [66] and Phillips-Perron [67] test to observe the order of integration. This study also used the Ng and Perron test which is considered as an effective modification of unit root tests. The inclusion of the PP test in this analysis was mainly due to its robustness to a set of time-dependent serial correlations and heteroscedasticities. The Akaike information criteria was used to evaluate the lag length in the ADF test case, while the Newey–West Bartlett kernel was used to select the bandwidth for the PP test. A conventional integration approach was applied and the null hypothesis generated suggested that the series was non-stationary, while it was stationary in the case of alternative hypotheses. The intercept and deterministic trends of time series data integrations were also used in this study.

### 3.1 Cointegration

Over the years, many cointegration methods have evolved. Engle and Granger [68] recommended a cointegration evaluation based on projected long-term regression model residuals. Several cointegration experiments were developed much later, such as Johansen’s system-based test [69], Boswijk’s ECM-based F-test [70], and Banerjee et al.’s ECM-based t-test [71]. These cointegration approaches have distinct theoretical bases and produce contradictory findings, thereby creating a limitation. The effectiveness of ranking cointegration techniques is significantly dependent on the value of nuisance estimators [72]. This study used a newly established cointegration test proposed by Bayer and Hanck [73] to verify the existence of cointegration relationship between environmental destruction and agricultural ecology in Bangladesh to solve the power of the cointegration tests. A distinctive feature of this test is that it helps integrate multiple individual findings from the test into a more decisive outcome. Bayer and Hanck [73] recommended combining the individual cointegration test’s computed significance level (p-values) with the following formulae postulated by Fisher (1 and 2):

(1) $$EG-J=-2\left[\ln\left(p_{EG}\right)+\ln\left(p_{J}\right)\right]$$

(2) $$EG-J-B-BDM=-2\left[\ln\left(p_{EG}\right)+\ln\left(p_{J}\right)+\ln\left(p_{B}\right)+\ln\left(p_{BDM}\right)\right]$$

Where p-values of several distinct cointegration tests such as Engle-Granger [69]; Boswijik [70] and, Banerjee et al. [71] are exhibited by P_EG_, P_J_, P_B_ and P_BDM_ respectively. Fisher statistics of the estimated values when exceeding the critical values proposed by Bayer and Hanck [73] for the null hypothesis of no cointegration can be rejected.

### 3.2 Long-run estimates

The Fully Modified Ordinary Least Squares (FMOLS) estimator developed by Phillips and Hansen [74] and the Dynamic Ordinary Least Square (DOLS) proposed by Stock and Watson [75] have several advantages over OLS estimators. FMOLS is mainly applied for a semi-parametric approach for long-run parameter estimation [76, 77]. FMOLS provides consistent estimator parameters [72, 78] and transforms data and parameters [72] in the case of small samples and succession over endogeneity or serial measurement error, omitting biased variables, solving heteroscedasticity, or serial correlation. FMOLS estimator Adom [79] is as follows (3):

(3) $${\hat{\beta }}_{FMOLS}={\left[\sum_{t=1}^{T}\left(x_{t}-\bar{x_{1}}\right)\left(x_{t}-\bar{x_{1}}\right)\right]}^{-1}*\left[\sum_{t=1\;}^{T}\left(x_{t}-\bar{x_{1}}\right)y_{t}^{*}+T{\tilde{△}}_{EM}\right]$$

$$y_{t}^{*}=y_{t}-\hat{{\text{Ω}}_{EM}}*{\text{Ω}}_{E}^{-1}\text{Δ}{\text{x}}_{t}$$

$${\tilde{\Delta }}_{EM}=\hat{{\text{Δ}}_{EM}{\text{Ω}}_{E}^{-1}{\text{Ω}}_{EM}}$$

Here, $\;y_{t}^{*}$ is the transformed variable of the dependent variable to attain the endogeneity equation, and ${\tilde{\Delta }}_{EM}$ is the serial correlation correction.

DOLS is an extension of the Stock and Watson’s [76] estimator, which is obtained from the following equation (4) -

(4) $$y_{t}=\alpha_{i}+\beta x_{t}+\overset{k}{\underset{j=1}{\sum }}c_{j}\Delta x_{t-j}+v_{it}$$

$c_{j}$ is the coefficient of a lead or lag and is the first differenced explanatory variable. The estimated coefficient of DOLS is given by the following equation (5):

(5) $${\hat{\beta }}_{DOLS}={\left[\sum_{t=1\;}^{n}q_{t}\;q_{t}\right]}^{-1}*\left[\sum_{t=1\;}^{n}q_{t}\;y_{t}^{*}\;\right]$$

Here, q is {2(q+1) ×1} vector of regressors and. $q_{t}=x_{t}-\bar{x_{i}}\Delta x_{t-q},\;\ldots \ldots ..\Delta x_{t+q}$

Canonical cointegrated regression (CCR), introduced by Park [80], works similar to FMOLS. However, there is a difference between these two models. CCR works on data transformation only, while FMOLS converts data and parameters [79, 80]. Further, CCR is also a single equation-based regression, which can also be applied to multivariate regression. The CCR estimator is obtained as follows (6) from Adom [79]:

(6) βˆCCR=∑t=1Txt-x1xt-x1-1∗∑t=1Txt-x1yt∗

### 3.3 Causality test

Changes in one variable may precede or follow changes in another variable, resulting in a variety of causal relationships. The objective of studying this causality is referred to as Granger causality (1969). The following models were studied to clarify the technique for analyzing Granger causality:

(7) $$Y_{t}=\overset{m}{\underset{i=1}{\sum }}a_{i}Y_{t-i}+\overset{m}{\underset{i=1}{\sum }}b_{i}X_{t-i}+u_{t}$$

(8) $$X_{t}=\overset{m}{\underset{i=1}{\sum }}c_{i}X_{t-i}+\overset{m}{\underset{i=1}{\sum }}d_{i}Y_{t-i}+v_{t}$$

Based on the above models (7 and 8), the following cases are distinguished:

• The coefficients b_i_ of the variables $X_{t-i}$ in (1) are statistically significant, while the coefficients c_i_ of $Y_{t-i}$ in (2) are statistically different from zero. In this case, there is Granger causality from X to Y.
• The coefficients b_i_ of the variables $X_{t-i}$ in (1) are not statistically significant, while the coefficients c_i_ of in $Y_{t-i}$ (2) are statistically different from zero. In this case, there is Granger causality from Y to X.
• The coefficients of b_i_ variables $X_{t-i}$ in (1) and c_i_ coefficients of $Y_{t-i}$ in (2) are statistically different from zero. In this case, there is bidirectional Granger causality.
• The coefficients of b_i_ variables $X_{t-i}$ in (1) and c_i_ coefficients of $Y_{t-i}$ in (2) are statistically different from zero. In this case, there is Granger independence.

Consequently, Granger causality estimation examines the null hypothesis that one variable does not cause another variable by examining the statistical significance of the estimated coefficients.

## 4 Results and discussion

The present study attempts to observe the causal relationship between agricultural production and carbon emissions by using time series data from the year 1985 till 2017. Results of basic statistical analysis along with their correlation analyses are tabulated in Table 2. It can be observed that the mean, median, and mode are close to each other following natural logarithmic transformation. Skewness describes the thickness or flatness, whereas kurtosis describes the peak nature of the distribution. From the results obtained in this study, it can be observed that CO_2_ emissions, the CO_2_ emission equivalent for N_2_O from synthetic fertilizer (SF) usage, and diverse crop production (OT) were all to the left. In the kurtosis analysis, none was distributed approximately, except for the CO_2_ emission equivalent that comes from synthetic fertilizer usage. Almost all the variables rejected normality under the Jarque-Bera statistic. Correlation describes the degree and direction of association amongst variables. Agricultural value-added contribution to the economy (AG) relates negatively to all variables except for other crop production (OT). Also, for CO_2_ emissions, this variable’s relationship was found to be more potent with burning of crop residues and biomass, agriculture machinery, cereal production, area harvested by rice production, and the CO_2_ emissions equivalent for N_2_O from synthetic fertilizers usage. A high correlation was observed among rice-harvested areas, cereal production, livestock, and agricultural machinery. Multicollinearity was also not observed.

**Table 2:** Basic statistics with correlation matrix of variables.

	CO_2_	BM	AG	SF	LS	MC	RI	CP	OT
Mean	6.761	15.652	3.104	8.661	16.664	8.654	16.18	17.427	12.988
Median	7.134	15.64	3.117	8.768	16.637	8.632	16.213	17.453	13.018
Maximum	8.23	15.732	3.547	9.051	16.874	8.838	16.3	17.876	13.412
SD	4.595	15.573	2.642	7.772	16.425	8.497	16.108	16.994	12.481
Skewness	1.188	0.051	0.279	0.334	0.134	0.102	0.061	0.299	0.207
Kurtosis	-0.491	0.213	0.075	-1.128	0.038	0.3	0.305	0.001	-0.439
Jarque Bera	1.897	1.741	1.657	3.412	1.89	1.815	2.092	1.567	2.847
Probability	3	2.429	2.512	7.231	1.703	2.425	1.646	2.822	1.094
Sum	0.223	0.297	0.285	0.027	0.427	0.297	0.439	0.244	0.579
Sum Sq. Dev.	223.115	516.528	102.417	285.818	549.926	285.576	533.949	575.085	428.602
Obs.	45.167	0.085	2.496	3.579	0.574	0.336	0.118	2.859	1.374
Correlations Matrix									
	CO_2_	BM	AG	SF	LS	MC	RI	CP	OT
CO_2_	1	0.725	-0.844	0.751	0.824	0.859	0.636	0.85	-0.198
BM	0.725	1	-0.813	0.633	0.782	0.839	0.852	0.884	-0.362
AG	-0.844	-0.813	1	-0.882	-0.968	-0.973	-0.767	-0.973	0.117
SF	0.751	0.633	-0.882	1	0.888	0.843	0.625	0.871	0.196
LS	0.824	0.782	-0.968	0.888	1	0.987	0.713	0.961	-0.049
MC	0.859	0.839	-0.973	0.843	0.987	1	0.748	0.975	-0.132
RI	0.636	0.852	-0.767	0.625	0.713	0.748	1	0.813	-0.344
CP	0.85	0.884	-0.973	0.871	0.961	0.975	0.813	1	-0.166
OT	-0.198	-0.362	0.117	0.196	-0.049	-0.132	-0.344	-0.166	1

### 4.1 Unit root test

A unit root test determines whether a time series component is non-stationary [81]. The results of unit root test presented in Table 3 highlights the effects of the ADF and the PP unit root tests. In both cases, the study variables were observed to be stationary at the first difference after logarithmic transformation, which was concurred to the findings of Ghimire et al. [82].

**Table 3:** Result of Augmented-Dickey Fuller (ADF) test and Phillips-Perron (PP) test.

Variable	ADF Test				PP Test			
	At level		At difference		At level		At difference	
	C	C + T	C	C + T	C	C + T	C	C + T
CO_2_	0.267	-1.975	-3.509∗∗∗	-3.306∗∗∗	-2.232	-3.215	-16.305∗∗∗	-15.691∗∗∗
BM	-0.056	-3.202	-3.012∗∗	-3.162∗∗∗	-0.921	-2.813	-6.620∗∗∗	-6.708∗∗∗
AG	0.062	-3.065	-5.549∗∗∗	-3.969∗∗∗	-0.051	-3∖2.095	-6.763∗∗∗	-6.643∗∗∗
SF	-2.749	-2.910	-4.554∗∗∗	-5.525∗∗∗	-5.570	-2.207	-6.393∗∗∗	-8.163∗∗∗
LS	-0.970	-1.891	-5.258∗∗∗	-5.219∗∗∗	-0.948	-2.143	-5.277∗∗∗	-5.242∗∗∗
MC	2.167	-1.957	-2.153	-3.707∗∗∗	1.158	-1.005	-2.819∗∗	-3.032∗∗∗
RICE	-1.974	-2.734	-7.479∗∗∗	-7.465∗∗∗	-1.974	-2.693	-8.129∗∗∗	-8.141∗∗∗
CP	0.296	-2.051	-4.030∗∗∗	-3.983∗∗	-0.391	-2.802	-5.460	-5.365∗∗∗
OT	-2.161	-3.249	-7.307∗∗∗	-0.614	-3.042	-2195	-6.787∗∗∗	-6.789∗∗∗

### 4.2 Ng-Perron test

This study also employed the Ng-Perron test for further investigation rather than relying only on the PP test and ADF test for stationarity testing. The goal of using this test was to show that Ng-Perron test was more powerful and more suggestive than the ADF test in the case of small sample size since the ADF test has been shown to be unreliable for small samples [83, 84]. The results of the Ng-Perron test (at level and at first difference) are presented in Tables Table 4a, Table 4b. From the results, it can be seen that only OT was weakly stationary at this level, but all were statistically significant at the first difference. As a result, it can be stated that at the first difference or integration, each variable is significant.

**Table 4a:** Result of Ng-Perron test (At level).

Variable	Ng-Perron Test							
	At level							
	Constant				Constant with trend			
	MZa	MZt	MSB	MPT	MZa	MZt	MSB	MPT
CO_2_	-2.690	-0.989	0.368∗	8.475∗	-23.990	-3.448	0.144	3.891
BM	-1.586	-0.645	0.407∗	11.33∗	-8.468	-2.044	0.241	10.803
AG	1.449	1.338	0.923	65.343	-14.028	-2.648	0.189	6.500
SF	0.119	0.089	0.749	35.161	-3.590	-1.213	0.338	23.335
LS	1.026	0.781	0.761	43.220	-7.271	-1.893	0.260	12.554
MC	-2.464	-0.793	0.322	8.401	-5.663	-1.611	0.284	15.923
RICE	-2.782	-0.844	0.303	7.820	-13.354	-2.536	0.190	7.089
CP	0.813	0.612	0.752	40.781	-15.360∗∗	-2.763∗∗	0.180	5.979
OT	-6.755∗∗∗	-1.773∗∗∗	0.262	3.847	-8.515	-2.057	0.242∗	10.721∗

**Table 4b:** Result of Ng-Perron test (At first difference).

Variable	Ng-Perron Test							
	At 1^st^ difference							
	Constant				Constant with trend			
	MZa	MZt	MSB	MPT	MZa	MZt	MSB	MPT
D(CO_2_)	-21.216∗	-3.253∗	0.153	1.169	-22.059∗∗	-3.321∗∗	0.151∗	4.132∗
D(BM)	-16.765∗	-2.869∗	0.171	1.557	-19.724∗∗	-3.115∗∗	0.158∗	4.775∗
D(AG)	-24.433∗	-3.49∗	0.143	1.021	-29.142∗∗	-3.806∗∗	0.131	3.190
D(SF)	-14.089∗	-2.653∗	0.188	1.743	-25.365∗	-3.541∗	0.140	3.714
D(LS)	-10.966∗∗	-2.325∗∗	0.212	2.299	-11.287	-2.374	0.210∗	8.083∗
D(MC)	-10.418∗∗	-2.279∗∗	0.219	2.364	-12.027	-2.449	0.204∗	7.592∗
D(RICE)	-6.951∗∗∗	-1.82∗∗∗	0.262	3.679	-13.183	-2.443	0.185∗	7.587∗
D(CP)	-23.729∗	-3.410∗	0.144	1.147	-23.648∗∗	-3.423∗	0.145∗	3.944∗
D(OT)	-23.629∗	-3.432∗	0.145	1.056	-25.338∗	-3.558∗	0.140	3.606

For Ng-Perron test, lag length is one. ∗, ∗∗, ∗∗∗ denotes the 1%, 5% and 10% level of significance, respectively.

### 4.3 Cointegration analysis

Fisher’s statistics were used in this study to observed if the underlying variables were cointegrated. The integrated cointegration measures, including the EG-JOH and EG-JOH-BO-BDM measures, are highlighted in Table 5. EG-JOH and EG-JOH-BO-BDM’s F-values were found to be greater than a critical value, implying that the null hypothesis of no cointegration can be rejected. A similar result was obtained by Chaouachi and Balsalobre-Lorente [85] in an earlier study. Therefore, Johansen and Bayer-Hanck’s cointegration tests confirmed that a long-run cointegration did occur between the selected variables in Bangladesh for the time period of 1985–2017.

**Table 5:** Cointegration result of Bayer-Hanck cointegration

Test statistic	EO-JOH	EO-JOH-BO-BDM	Decision
F-statistic	58.413	168.934	Cointegration Analysis
Critical regions			
1%	15	19.899	
5%	10.181	19.447	
10%	8.134	15.595	

Bayer and Hanck [73] developed a combined cointegration approach that strengthens the cointegration analysis. This innovative technique amalgamates findings of previous cointegration and depicts on Fisher F-statistic with consistent and decisive results. However, strong prohibition is needed of this hypothesis-based cointegration i.e. the order of each variable is stationary at first difference. An F-value greater than a critical value indicates that the null hypothesis of no cointegration should be rejected and F-values less than the critical value accepts the null hypothesis of no cointegration. Value of F-statistic under 1%, 5%, and 10% level of significance, both statistic is higher than critical values. Therefore, there exists a cointegration among variables.

### 4.4 Long run coefficients

As the variables were integrated at first order, the cointegration in this study was examined through FMOLS, DOLS, and CCR. Carbon dioxide emission is negatively associated with biomass-burned crop residues, agricultural land added to GDP, livestock, rice harvested area, cereal production, and other production in three models. From the data in Table 6, it can be seen that in FMOLS, the negative effect of CO_2_ emissions is significantly related to crop residues and biomass burning, livestock, rice harvested on land area, cereal production, and other crop production. This is because the more agricultural production there is, the more is the demand for use of combustible energy resources which consequently leads to release of emissions into the environment. This finding is similar to that of the studies conducted prior [86] and [87] in four ASEAN countries and in another study conducted [88] for E7 countries. CO_2_ emissions are pushed up a lot by the CO_2_ equivalent of nitrous oxide (N_2_O) emissions from synthetic fertilizers and other farming equipment [89]. In the case of DOLS, the results indicated that livestock and other crop production decreased CO_2_ emissions. All variables’ effects were statistically associated with CO_2_ emissions except crop residues and biomass burning in CCR. A study by Adedoyin et al. [88] found that energy use in the form of renewable energy had a negative and significant coefficient at various levels of significance. In the E7 countries, a 1% increase in energy use resulted in a 0.32 to 0.66 percent reduction in CO_2_ emissions [88].

**Table 6:** Cointegrated coefficients of variables under FMOLS, DOLS and CCR.

Variable	FMOLS				DOLS			CCR		
	Coefficient	t-statistic	p-value	Coefficient	t-Statistic	p-value	Coefficient		t-Statistic	p-value
BM	-2.295	-2.813	0.010	-6.017	-1.181	0.249	-0.866		-0.868	0.394
AG	-0.009	-0.034	0.973	1.314	0.801	0.431	-1.416		-4.208	0.000
SF	2.195	12.974	0.000	3.238	3.354	0.003	1.293		4.725	0.000
LS	-23.577	-24.683	0.000	-23.870	-4.037	0.001	-15.000		-14.552	0.000
MC	39.786	25.627	0.000	41.840	4.441	0.000	27.863		14.727	0.000
RICE	-2.444	-5.274	0.000	-1.405	-0.498	0.623	-1.666		-3.768	0.001
CP	-1.539	-4.061	0.001	-1.302	-0.566	0.576	-1.825		-2.877	0.009
OT	-0.630	-6.699	0.000	-1.365	-2.325	0.029	-0.481		-3.718	0.001
Constant	146.910	11.000	0.000	167.677	2.015	0.055	87.596		6.209	0.000

A causal connection was observed in Bayer-Hanck’s cointegration. The Granger Causality test [90] was used to examine pairwise relationships. The results are presented in Table 7. Significant impact was observed on the agricultural value added to GDP when it comes to burning of biomass and crop residues and cereal production as well as environmental degradation and agricultural machinery. There was also a significant impact on the rice harvested area. Some unidirectional causality was observed from crop production to crop residues and biomass burning, livestock, rice harvested area, and other production (CP→LS, CP→RI, CP→BM, CP→OT). The results obtained in this study suggested that crop production was significantly affected by agricultural machinery, livestock plowed, rice cultivation, and other cereal production. Unidirectional causality was observed for CP→CO_2_, AG→CO_2_, MC→CO_2_, CP→CO_2_ & CP→CO_2_, LS→BM, MC→BM, AG→BM, SF→RI, AG→RI, CP→RI, MC→RI, and so on.

**Table 7:** Granger causality test.

Null Hypothesis	F-Statistic	p-value	Null Hypothesis	F-Statistic	p-value
AG does not Granger Cause CO_2_	8.708	0.001	AG does not Granger Cause RI	11.107	0.000
LS does not Granger Cause CO_2_	5.370	0.011	AG does not Granger Cause CP	12.374	0.000
CO_2_ does not Granger Cause LS	6.719	0.004	AG does not Granger Cause OT	4.269	0.025
MC does not Granger Cause CO_2_	12.702	0.000	SF does not Granger Cause RI	4.792	0.017
RI does not Granger Cause CO_2_	4.084	0.029	MC does not Granger Cause LS	6.483	0.005
CO_2_ does not Granger Cause RI	3.934	0.032	LS does not Granger Cause RI	4.616	0.019
CP does not Granger Cause CO_2_	8.436	0.002	CP does not Granger Cause LS	7.651	0.002
AG does not Granger Cause BM	7.048	0.004	RI does not Granger Cause MC	3.444	0.047
LS does not Granger Cause BM	3.436	0.047	MC does not Granger Cause RI	5.346	0.011
MC does not Granger Cause BM	3.526	0.044	MC does not Granger Cause OT	7.800	0.002
CP does not Granger Cause BM	4.604	0.019	CP does not Granger Cause RI	7.548	0.003
LS does not Granger Cause AG	7.774	0.002	RI does not Granger Cause OT	4.314	0.024
AG does not Granger Cause MC	7.203	0.003	CP does not Granger Cause OT	3.460	0.047
Null Hypothesis	F-Statistic	p-value	Null Hypothesis	F-Statistic	p-value

CO_2_ emissions and/or environmental degradation caused a 5% level of significance with livestock and rice harvested area in a bidirectional causality (CO_2_↔LS, CO_2_↔RI), which was similar to the findings of an earlier study [91], and suggests that as livestock increases, the chance of environmental degradation also increases. Rice is one of the staple foodgrains and the area where rice paddy fields are cultivated have a direct link to carbon emissions. Meijide et al. [92] claim that rice paddy fields are fundamental indicators of anthropogenic CO_2_ emissions. Environmental degradation forces causal linkage of two factors into unidirectional causalities, such as agricultural machinery and cereal production (MC→CO_2_ & CP→CO_2_). Similar findings were also observed by Ullah et al. [60]. Therefore, it can be stated based on the observed findings that tillage and equipment used in an excessive manner can potentially stimulate environmental degradation. In Canadian firm, agricultural machinery and fuel play a role in CO_2_ emissions [93]. Unlike the findings of [94] and [95], there was no causal evidence of crop residue and biomass burning with CO_2_ emissions increase. Crop production has been shown to cause CO_2_ emissions increase in a unidirectional causality for Ghana [39]. Therefore, crop production can also positively attribute to carbon emissions. The monotonous increasing trend of environmental degradation can hamper the ecological balance. This onset triggers climatic hazards resulting from floods, cyclones, temperature increases, precipitation imbalances, and production in cereal areas being disrupted [96]. The findings of that study was opposite to that of Pant [97] who proposed that rising CO_2_ can be utilized for agricultural productivity via a combination of accelerated photosynthesis and water use efficiency.

Several previous studies have studied the nexus between agriculture value addition, energy use, economic growth, and emissions in the context of different countries [60, 88, 98, 99, 100, 101]. However, this study differs from the existing literature by complementing it and considering the relationship between agricultural ecosystems and Bangladesh’s environmental degradation from 1985 to 2017. Consequently, this study would be a major step forward in environmental and agricultural research. The findings of this study establishes a bidirectional causal relationship between environmental degradation and livestock, as well as between environmental degradation and rice harvest areas. This finding was previously supported by Owusu and Asumadu-Sarkodie [91], who discovered bidirectional causality between milled rice production and carbon dioxide emissions in their study. The results obtained in this study showed that environmental degradation had a one-way causal relationship with agricultural machinery and with cereal production. Another study in Pakistan [48] found similar results, stating that burning crop residues, livestock, farm equipment, cereal production, and other crop products all contribute significantly to CO_2_ emissions. Finally, the findings of the current study indicated that livestock, cereal production, agricultural machinery use, and agricultural land production all contributed to carbon dioxide emissions. Due to high land fragmentation and population growth, Bangladesh’s cultivable land area has decreased over time, putting enormous pressure on the country’s limited land resources to produce enough food for sustaining the growing population.

## 5 Limitations of the current study and scope for future research

Though there are various sources of anthropogenic emissions, this study focused only on the emissions resulting from agricultural production and agro-economy of Bangladesh. To achieve the proposed UNSDGs by 2030, any country must pay attention to social, economic, and environmental aspects. This study may support a two dimensional idea for policymakers to prepare for any future strategies. Moreover, the findings obtained in this study may provide an opportunity for future research to compare the situation of Bangladesh with that of other similar nations in order to identify whether obstacles are unique to all nations or vary from nation to nation. One limitation of this study was the lack of availability of most recent data and hence considered the datasets available until 2017.

## 6 Conclusions and policy implications

This study was confined to analysing the agricultural ecosystem from 1985 to 2017. On the other hand, rice as a staple food is considered in this study as a harvested area. Researchers consider more variables like cereal and other production, biomass burned crop residues, and agricultural machinery for modern technology to tackle the agroecosystem. The present study applied two customary unit root tests to study variables and concluded that each variable is stationary at I (1). Cointegration was confirmed by the Bayer-Hanck cointegration at a 1% level of significance. In particular the analysis of variable in FMOLS, DOLS, and CCR evidenced that environmental degradation is accelerated with a 1% significance in long-run estimates. A pairwise Granger Causality test was computed to check out causality with the results concluding that carbon dioxide emissions were caused by cereal production, agricultural production, and agricultural land production. Therefore, farming in Bangladesh is still considered to be causing environmental degradation due to unplanned and stagnant crop intensity combined with a lack of modern facilities. Due to high land fragmentation and a growing population, the arable land area of Bangladesh is declining, thereby creating huge pressure on limited land resources to produce sufficient food for all. To meet the nation’s food requirements, the agricultural sector initiates such a method that is harmful to nature as well as our survival on earth. On the other hand, environmental degradation is accelerated due to unnecessary or unplanned farming of agricultural land, tillage use, lack of technological innovations and poor water management, primitive-based farming, securing employment, and raising entrepreneurialism from foreign investors and banking sides. This research envisages to contribute to a strong economic growth and a high-quality environment, keeping in line with the UN-SDG Targets of 7, 9, 12, and 13 on clean energy access and climate change mitigation. The majority of Bangladeshi farmers are illiterate and unaware of environmental issues. As a result, they harm nature both purposefully and accidently. Furthermore, most of the Bangladeshi population is not sufficiently conscious of environmental degradation, and there is a lack of environmental concern at every societal level. However, pollution can be reduced by implementing appropriate policies, laws, initiatives, and effective implementations. The findings of this study reveal that environmental contamination has a strong causal association with Bangladesh’s agricultural ecology. By reinforcing and adapting the existing policies and strategies, the Government of Bangladesh should introduce some synchronized policies that may help achieve a sustainable agro-economic system by reducing environmental pollution. The following are some recommendations.

• Increasing subsidies and government spending from a fiscal policy standpoint should result in certain projects that match demand.
• Establishing watchdog bodies to oversee the overall agriculture activities and identify the farmers responsible for creating more natural degradation.
• The use of harmful organic and inorganic products such as pesticides and chemical fertilizers should be reduced by developing awareness among farmers for this purpose and promoting biological evolution through using organic fertilizer.
• Technological advancement should be used to make the necessary stakeholders aware of the benefits of sustainable agriculture and protecting the environment.
• Though the farmers of Bangladesh are poor, setting penalties for fixed level of emissions transgressions may decrease environmental degradation.
• For a more robust agrarian economy, policies must be more aggressive for bridging the demand gap created by disasters or calamities that have devastated the low-lying lands.

## Declarations

### Author contribution statement

Shanjida Chowdhury: Conceived and designed the experiments; Analyzed and interpreted the data; Wrote the paper.

Sunjida Khan; Md Fouad Hossain Sarker; Md Kabirul Islam: Performed the experiments; Wrote the paper.

Maruf Ahmed Tamal: Analyzed and interpreted the data; Wrote the paper.

Niaz Ahmed Khan: Conceived and designed the experiments; Wrote the paper.

### Funding statement

This research did not receive any specific grant from funding agencies in the public, commercial, or not-for-profit sectors.

### Data availability statement

Data will be made available on request.

### Declaration of interest's statement

The authors declare no conflict of interest.

### Additional information

No additional information is available for this paper.
